# Care of Geriatric Patients with Lumbar Spine, Pelvic, and Acetabular Fractures before and after Certification as a Geriatric Trauma Center DGU^®^: A Retrospective Cohort Study

**DOI:** 10.3390/medicina57080794

**Published:** 2021-07-31

**Authors:** Tobias Hafner, Alina Kollmeier, Markus Laubach, Matthias Knobe, Frank Hildebrand, Miguel Pishnamaz

**Affiliations:** 1 Department of Trauma and Reconstructive Surgery, RWTH University Hospital, 52074 Aachen, Germany; alina.kollmeier@rwth-aachen.de (A.K.); mlaubach@ukaachen.de (M.L.); fhildebrand@ukaachen.de (F.H.); mpishnamaz@ukaachen.de (M.P.); 2 Department of Orthopedic and Trauma Surgery, Cantonal Hospital, 6004 Lucerne, Switzerland; matthias.knobe@luks.ch

**Keywords:** ortho-geriatric co-management, elderly, geriatric trauma center, DGU

## Abstract

*Background:* More than 750,000 fragility fractures occur in Germany every year, with an expected increase in the following years. Interdisciplinary care pathways for geriatric patients are increasingly established to improve the treatment process and outcome, but there has been only limited evaluation of their use. Objectives: This study aimed to compare patient care before and after the implementation of a geriatric trauma center (GTC) in conformity with the German Society for Trauma Surgery (DGU^®^). *Patients and Methods:* We performed a retrospective single-center cohort study, including 361 patients >70 years old with lumbar spine, pelvic, and acetabular fractures, admitted between January 2012 and September 2019. Patients were divided into a usual care cohort (UC, *n* = 137) before implementation and an ortho-geriatric care cohort (OGC, *n* = 224) after implementation of the GTC DGU^®^. We recorded and compared demographic data, fracture type, geriatric assessment and management, therapy, complications, and various clinical parameters, e.g., length of stay, time to surgery, hours admitted to ICU, and change in walking ability. *Results:* The geriatric assessment revealed significant geriatric co-morbidities and a need for geriatric intervention in 75% of the patients. With orthogeriatric co-management, a significant increase in the detection of urological complications (UC: 25.5% vs. OGC: 37.5%; *p* = 0.021), earlier postoperative mobilization (UC: 57.1% vs. OGC: 86.3%; *p* < 0.001), an increased prescription of anti-osteoporotic treatment at discharge (UC: 13.1% vs. OGC: 46.8%; *p* < 0.001), and lower rates of revision surgery (UC: 5.8% vs. OGC: 3.1%; *p* = 0.012) could be seen. *Conclusions:* Our results emphasize the improvement in patient care and clinical outcome by implementing a GTC DGU^®^ and provide opportunities for future improvement in ortho-geriatric patient care.

## 1. Introduction

Fractures in the geriatric population are becoming more important in Europe and other Western countries due to the demographic change and increasing mobility in older patients. This causes a high individual and socioeconomic burden [1]. The number of fragility fractures in Germany in 2017 was estimated at 765,000, with an expected increase of 18.5% by the year 2030 [2]. The growing population of geriatric fracture patients brings new challenges in patient care, as they are highly vulnerable to medical complications, such as delirium, pressure ulcers, or infections due to a variety of pre-existing comorbidities, including malnutrition, cardiovascular diseases, or cognitive impairment [3]. It has been extensively demonstrated that these comorbidities correlate with increased mortality and worse clinical outcomes [4,5].

Therefore, geriatric patients require adapted and more demand-oriented care, which goes beyond the mere treatment of the fracture sustained. The medical field of geriatrics is specialized in identifying and treating patients with high-risk profiles and vulnerability. Over the past few decades, synergies between trauma/orthopedic surgery and geriatrics have thus developed in response to the changing requirements in patient care. This so-called ortho-geriatric co-management aims not only at the prevention and treatment of acute medical complications, but also at the long-term restoration of functionality and autonomy [6]. While there have been conflicting results on the outcome of ortho-geriatric co-management in the early years [7], numerous studies recently demonstrated a reduction in complications, readmission rates, and mortality [8,9,10]. In addition, as the economic burden of patient care is getting more important, the advantages of such models were analyzed and highlighted by several working groups [11,12]. 

Briefly, there are mainly three different models of co-management: (i) admission to an orthopedic or trauma surgery ward with the routine consultation of a geriatrician [9,12]; (ii) admission to a geriatric ward with regular consultation of an orthopedic or trauma surgeon [13]; and (iii) an integrated care model with shared responsibility between geriatrics and trauma surgery [10,14]. The substantial superiority of a single model has not been determined in the past [6]. 

In Germany, the concept of a geriatric trauma center (AltersTraumaZentrum DGU^®^) in combination with an age trauma registry (AltersTraumaRegister DGU^®^), enabling interdisciplinary treatment according to evidence-based and standardized criteria, was presented for the first time in 2013 [15]. Since then, 108 GTCs certified by the German Society for Trauma Surgery (DGU^®^) have been established in Germany, Austria, and Switzerland. However, scientific evaluation regarding the need, effectiveness, and outcome of these centers in Germany and other countries worldwide is mostly limited to geriatric patients with hip fractures, as these are the leading fracture type in quantity, mortality, and economic burden [11,12]. Since the numbers of pelvic and spine fractures are rapidly increasing [16,17] and comorbidities and treatment pathways differ between the types of fractures, it is necessary to investigate a broader spectrum of fractures and evaluate them in the context of ortho-geriatric co-management.

Therefore, the purpose of this study was to evaluate the necessity of a GTC and its impact on improving the care and clinical outcome of patients with lumbar spine, pelvis, and acetabular fractures. In particular, we tried to answer the question if the establishment of standardized ortho-geriatric co-management in conformity with the requirements of the DGU^®^ resulted in (i) improved clinical outcomes and lower rates of complications, and (ii) in a more demand-oriented care for older patients.

## 2. Patients and Methods

The local institutional review board approval (ethical committee, RWTH Aachen, Germany, AZ-EK 284/16) was obtained prior to the study. This is a retrospective single-center cohort study based on data collected from the electronic patient records at the university hospital of Aachen, Germany. According to the definition of a geriatric patient by the German Society for Geriatrics [18], all geriatric patients aged > 70 years who were admitted with lumbar spine, pelvic, or acetabular fractures in the period from January 2012 to September 2019 were included. Polytrauma patients (Injury Severity Score ≥ 16), tumor-associated fractures, and patients treated only in the ICU were excluded.

Certification as a GTC DGU^®^ was achieved in January 2015, resulting in a pre-certification usual care (UC) cohort from January 2012 to December 2014 (*n* = 137) and a post-certification ortho-geriatric co-management (OGC) cohort from January 2015 to September 2019 (*n* = 224).

The ortho-geriatric model implemented in this study is based on the ward round model in conformity with the certification guidelines of the DGU^®^ [12,19]. Patients were admitted to and treated at the trauma surgery ward with the routine consultation of a geriatrician in an interdisciplinary ward round twice a week and an interdisciplinary team conference once a week. Moreover, representatives of nursing, occupational therapy, physiotherapy, and case management took part in the treatment process from admission until discharge. All participants were advised to provide care following the standard operating procedures (SOPs) provided by the DGU^®^ for geriatric trauma patients [12].

For all patients, basic demographic data and the American Society of Anesthesiologists (ASA) classification were collected. The fracture type was grouped into either (i) lumbar spine, (ii) pelvis, or (iii) acetabulum fracture. Multiple fractures of the lumbar spine and pelvis, as well as fractures of other and unspecified parts of the lumbar spine and pelvis, were combined into a fourth group of others. Information on the presence and severity of comorbidities was obtained by calculating the age-adjusted Charlson Comorbidity Index (ACCI) [20].

After the establishment of the GTC, a geriatric assessment was performed with patients of the OGC cohort. This included the Identification of Seniors at Risk (ISAR) screening [21], the Barthel Index (BI) [22], the modified Barthel Index (mod. BI) according to Prosiegel et al. [23], the Geriatric Depression Scale (GDS) [24], the De Morton Mobility Index (DEMMI) [25], the Mini Nutritional Assessment (MNA) [26], the Montreal Cognitive Assessment (MoCA) [27], and the Dementia Detection Test (DemTect) [28]. In addition, the co-management with a geriatrician and the time from admission to the initial contact with a geriatrician was obtained.

As the primary outcome measures, acute kidney injury, anemia, delirium, dehydration, electrolyte disorders, surgical complications, complications during anesthesia, revision surgery, an in-hospital fall, transfusion of blood products, sepsis/SIRS/shock, and in-hospital mortality were collected. In addition, a group-wise assessment of cardiac, pulmonary, gastrointestinal, urologic, and neurologic complications was performed.

As secondary outcome measures, therapy regimen (surgery vs. conservative), time to surgery (hours), hours admitted to the ICU, length of stay (days), mobilization on the first postoperative day, anti-osteoporotic treatment at discharge, and pressure ulcers at discharge were obtained. Furthermore, the walking ability at discharge was determined in relation to the walking ability prior to the fracture. Therefore, walking ability before the fracture event and at discharge were coded numerically as followed: (1) = no functional walking ability; (2) = with a walking frame without wheels or an assistant; (3) = with a walker or two crutches; (4) = with a walking stick or one crutch; (5) = without assistance. By subtracting the value before the fracture event from the value at discharge, an improved or reduced walking ability could be displayed by a negative or positive result, respectively. 

All statistics were performed using IBM SPSS Statistics (Version 25; IBM, New York, NY, USA). Normal distribution was checked by the Shapiro–Wilk test and the Kolmogorov–Smirnov test with Lilliefors correction. Non-normally distributed and unpaired data with metric and ordinal variables (i.e., age, BMI, ACCI, in-hospital mortality, length of stay, time to surgery, hours admitted to ICU, and change in walking ability) were compared using the Mann–Whitney U test. For ordinal, alternative, and categorical data, the asymptotic significance was reported. Data with categorical variables (i.e., sex, ASA, fracture type, medical complications, therapy regimen, pressure ulcer, suspected dementia, anti-osteoporotic treatment at discharge, mobilization at first postoperative day) were compared using the chi-squared test (χ^2^) or Fisher test, if appropriate. Qualitative data are expressed in absolute (*n*) and relative (%) frequencies, and quantitative data are given as the mean ± standard deviation. The level of significance was set to *p* < 0.05. 

## 3. Results

### 3.1. Demographics

The demographic characteristics are shown in Table 1. A total of 361 patients were included, of which 137 were assigned to the UC cohort and 224 to the OGC cohort. The mean age was 81.9 ± 6.2 years with no significant difference between the cohorts (*p* = 0.566). In terms of sex distribution, the two cohorts did not display significant differences (*p* = 0.326), with both cohorts having a substantially higher proportion of female patients (UC: 74.5% vs. OGC: 68.6%). Statistically significant differences were seen in the patients’ ASA classification; a larger number of patients in the OGC cohort were assigned to ASA group III/IV (UC: 68.8% vs. OGC: 75.4%; *p* = 0.002). Moreover, the two cohorts differed significantly regarding the fracture entities. While pelvic fractures were the most common fracture entity in the OGC cohort (43.8%), they ranked second in the UC cohort with 37.2%, after lumbar fractures, with 40.1%. However, a post hoc test revealed that statistical significance was limited to the subgroup of others only (UC: 6.6% vs. OGC: 0.4%; *p* < 0.001). 

### 3.2. Geriatric Assessment and Co-Management

The ISAR screening, defining patients with a need for geriatric treatment, was performed on 87.5% of all patients in the OGC cohort, with a mean score of 2.9 ± 1.7 (Table 2). 

When testing the skills of daily living to assess the patients’ dependency, the BI averaged 39.2 ± 16.5 points, which indicated a severe dependency of all patients in the activities of daily living. In the mod. BI (*n* = 79) and the MoCA (*n* = 30), quantifying the patients’ cognitive abilities, scores of 71.3 ± 21.7 and 22.9 ± 4.4 were achieved, respectively. More specifically, 34.2% of the patients showed at least moderate cognitive impairment in the mod. BI (mod. BI ≤ 65), and 63.3% of the patients demonstrated cognitive impairment in the MoCA (MoCA < 26). Screening for depression using the GDS (*n* = 95), an average score of 3.6 ± 2.4 was obtained, with 14.7% and 2.1% showing signs of mild and severe depression, respectively. Furthermore, 17.5% of the patients were tested for signs of dementia by the DemTect, with an average score of 6.9 ± 4.2. Of these patients, 69.2% were suspected of having dementia (DemTect < 8). 

The DEMMI test revealed an average score of 23.4 ± 10.2, and was performed on 32.1% (*n* = 72) of the patients, while the MNA (*n* = 109) led to an average score of 21.2 ± 4.4, indicative of a risk for malnutrition in the majority of the patients (MNA 17–23.5). Only 31.2% showed a healthy nutritional status (MNA ≥ 24). For a detailed subdivision of the data presented, please see Supplementary Table S1. 

Moreover, the electronic patient records revealed that in 54.5% of the 224 cases, the patients were co-managed by a geriatrician, and it took an average of 95.9 ± 70.5 h until the first contact with the geriatrician.

### 3.3. Primary Outcome Measures

Concerning the clinical complications (Table 3), we observed a significantly higher rate of detected urological complications after the establishment of the GTC (UC: 25.5% vs. OGC: 37.5%; *p* = 0.021). A similar unambiguous trend was seen in the group of pulmonary complications (UC: 12.4% vs. OGC: 17.9%; *p* = 0.071), as well as in the identification of delirium (UC: 6.6% vs. OGC: 12.1%; *p* = 0.091), although statistical significance was not reached. 

The establishment of ortho-geriatric co-management resulted in significantly fewer numbers of revision surgery required (UC: 5.8% vs. OGC: 3.1%; *p* = 0.012). The reasons for revision surgery included wound infection, hip dislocation, mechanical complications, the need for lumbar cage implantation, and subsequent laminectomy. This contrasted with the trend towards lower surgery complications in general in the UC cohort (2.2%) compared to the cohort after GTC establishment (5.4%) (*p* = 0.143). The further complications, e.g., dehydration, electrolyte disorder, complications during anesthesia, and in-hospital mortality, did not reveal any significant differences between the two cohorts. 

### 3.4. Secondary Outcome Measures

While the total hospital length of stay remained unchanged after implementation of the GTC, the time to surgery significantly increased from 72.4 ± 54.5 h to 112.2 ± 45.5 h (*p* < 0.001) (Table 4). After the establishment of the GTC, the documentation of suspected dementia in the patient records significantly increased from 0.7% to 5.8% (*p* = 0.022). Similarly, the establishment of osteoporosis treatment at discharge was significantly increased (*p* < 0.001). It should be noted that four individuals in the OGC cohort had conflicting information regarding osteoporosis therapy, and were thus excluded from the calculation. Information on mobilization on the first day after surgery was available in 84 and 117 cases in the UC and OGC cohorts, respectively. Thereby, a significantly higher number of patients being mobilized was observed after the establishment of the GTC (UC: 57.1% vs. OGC: 86.3%; *p* < 0.001). When comparing the walking ability at discharge with the walking ability before the fracture event, a slight decrease in walking ability was observed in both cohorts (UC: −0.92 ± 1.38 vs. OGC: −0.64 ± 1.07; *p* = 0.252). However, a higher number of patients with restored or improved walking ability at discharge could be seen in the OGC cohort (UC: 52.4% vs. OGC: 63.9%).

## 4. Discussion

Demographic changes and the associated increase in older patients with fractures require new approaches for therapy and patient care. To overcome highly heterogenous models of ortho-geriatric care, certified GTCs with standardized, interdisciplinary ortho-geriatric co-management have been established under the stewardship of the DGU. In this retrospective cohort study, we investigated the clinical outcome and effectiveness of patient care after the establishment of a geriatric trauma center DGU^®^ at a German university hospital. While most studies have evaluated hip fractures as the most common fragility fracture in the context of GTCs [9,10,14,29], this is one of the first studies to focus on patients with pelvic, lumbar spine, and acetabular fractures. 

We were able to show that the conceptual implementation of a GTC DGU^®^ (i) improves the clinical outcome by reducing the rate of revision surgeries and increased identification of urological complications, and (ii) leads to a more demand-oriented patient care beyond the actual fracture management, e.g., by improving osteoporosis prophylaxis or earlier postoperative mobilization. We note that, due to differences in national healthcare systems, the design and extent of implementation of ortho-geriatric co-management may vary, and therefore, in some cases, affect comparability with the clinical outcomes observed in the present work. However, there is good comparability with previous results published by other groups reporting on patients treated in a DGU-certified GTC.

### 4.1. Geriatric Assessment

The geriatric assessment is a time-consuming but essential part of ortho-geriatric co-management to identify patients at risk, establish an individual risk profile, and thus establish a demand-oriented treatment protocol for each patient. In this context, an ISAR score ≥2 is seen as an indication for geriatric co-treatment, and is one of the quality criteria of a GTC DGU^®^ [21]. In this study, the screening was performed in 89.5%, and the cutoff score (≥2) was reached by 75%, proving a need for geriatric involvement in the majority of our patient population. Thereby, these results are in line with previous traumatological studies demonstrating a rate of ISAR screening ≥2 in 71% to 81% of the patients [30,31]. However, to avoid overtreatment on the one hand and missing patients at risk on the other hand, a screening rate of 100% should be aimed for in the future.

The high-risk profile of the patients in this study is emphasized by the results of the further geriatric assessment. All patients tested showed a limited physical ability in the BI (39.2 ± 16.5). The majority also exhibited cognitive impairment, assessed by the mod. BI (71.3 ± 21.7), the MoCA (22.9 ± 4.4), and the DemTect (6.9 ± 4.2). Even if the prevalence of cognitive impairment and BI scores differ widely in geriatric studies [32,33], their use is of great importance, as increased BI and cognitive impairment have been shown to correlate with increased mortality and reduced long-term survival [34]. The same applies to the identification of malnutrition. By using the MNA, a risk of malnutrition could be observed in 53.2% and manifest malnutrition in 15.6%. After the identification of patients at risk, perioperative optimization of the nutritive situation is capable of preventing complications and improving the quality of life [35]. 

Unfortunately, most ortho-geriatric studies do not allow a definite conclusion on the actual impact of performing a geriatric assessment on the clinical outcome. A systematic Cochrane review by Eamer et al., however, observed a reduction in mortality, length of stay, and medical costs by implementing a comprehensive geriatric assessment before the actual treatment [36]. The co-treatment by a geriatrician in our study was provided in 54.5%, and it took an average of 95.9 ± 70.5 h until the initial contact with the geriatrician. These results are undoubtedly below our expectations. As most comparable studies rely on a daily visit by the geriatrician [37,38] or are based on shared responsibility for patient care, comparison here is limited. However, reasons for the time delay and the low number of co-treatment could be a delayed geriatric screening, e.g., due to admission on weekends, absence of the patient during the ward round, or ICU admission. Moreover, in the course of the observation period after the establishment of the GTC, an ISAR score ≥2 upon admission was introduced as a new criterion for co-treatment by a geriatrician. The aim thereby was to avoid unnecessary overtreatment. Since then, patients with ISAR <2 did not receive geriatric co-treatment, but were still included in the study to ensure continuity. This also could explain the low rate of co-treatment by the geriatrician. Nevertheless, earlier contact and a higher rate of co-treatment by a geriatrician will be necessary in the future. 

### 4.2. Primary Outcome Measures

Ortho-geriatric co-treatment significantly increased the numbers of detected urological complications from 25.5% to 37.5%. A similar trend could be seen in pulmonary complications and delirium. Thus, our results contradict previous studies, which predominantly observed a reduced detection of the aforementioned complications [9,11]. Fisher et al., for example, were able to demonstrate a reduction in delirium from 11.7 % to 5.9% and a reduction in urinary tract infections from 8.9% to 6.7% in the co-care of older patients with hip fractures [9]. Our results must also be considered in the context of the higher ASA scores of the OGC cohort, which indicate poorer baseline health conditions. Thus, in addition to an increased detection rate, a possible increase in actual complications cannot be entirely dismissed. Therefore, the interpretation of complication rates in co-management is not unambiguous, as ortho-geriatric co-management aims to decrease the overall risk of complications by preventive actions on the one hand, but concomitantly can lead to higher rates by a more focused clinical examination and strict monitoring of early signs of complications on the other hand. As such, Knobe et al. observed a trend towards an increase in delirium after the establishment of a GTC from 20% to 26%, as well as an increase in urinary tract infections and renal complications [12]. Accordingly, Folbert et al. demonstrated an increase in the diagnosis of delirium after establishing their GTC [29]. The mere consideration of the complication rate, therefore, does not seem to be appropriate in this context, as it does not allow to distinguish between an increased detection rate and an actual reduction of the complication. Rather, an evaluation of the complications in their consequence on the course of treatment, for example by using the adapted Clavien−Dindo scoring system [39] should be included in the future. Nevertheless, the rate of revision surgeries, which unquestionably is related to a high risk for older patients, could be significantly reduced, although the total number in both cohorts can be considered low. Concomitantly, the incidence of anemia and the required transfusions of blood products were reduced from 26.3% to 21.0% and from 21.2% to 17.9%, respectively. These results underline the positive impacts of our GTC DGU^®^ on the clinical outcome.

A frequently used parameter in the evaluation of patient care and treatment is the reduction of in-hospital mortality. With the intensification of orthogeriatric management, decreasing mortality rates could be observed in the past [10,12,14,37]. In contrast, our results showed a slight increase in in-hospital mortality from 0% to 2.7%. One possible reason for the increased mortality in the OGC cohort may again be the poorer baseline health conditions, represented by the higher ASA scores. Thereby, it should be noted that mortality rates of 0% and 2.7%, respectively, are significantly lower than the average mortality rates in previous studies. For example, Grund et al. demonstrated mortality rates of 9.5% and 6.5 %, respectively, whereas Pablos-Hernández et al. observed a reduction from 5.1% to 3.4% [14,40]. Up to now, the literature on in-hospital mortality with ortho-geriatric co-management remains inconclusive [15]. Overall, an unambiguous comparison of complications and clinical outcome parameters between previously published studies often remains difficult due to substantial differences in national health systems, ortho-geriatric treatment models, and definitions of complications [6,41]. More studies using the same standardized and certified model as the GTC DGU^®^ will be necessary to evaluate their influence on the reduction of complications and clinical outcome.

### 4.3. Secondary Outcome Measures

Both cohorts revealed a prolonged time to surgery, with a significant increase after the establishment of the GTC from 72 to 112 h. This is mainly due to the fact that surgical treatment of pelvic and spine fractures is often preceded by an attempt of non-surgical therapy. Only if pain-free mobilization is not achieved is surgical stabilization of the fracture considered. In comparison with other studies, it should be noted that the majority of ortho-geriatric studies focused on hip fractures that require surgical treatment within the first 24 h [37,41]. In a study with a broader spectrum of fractures [12] that additionally included spine, pelvis, and acetabular fractures, an average time to surgery of 40 h was reported. The extended time to surgery in the OGC cohort does not necessarily imply inadequate patient care. Rather, the poorer baseline health conditions, as well as the intensive geriatric assessment, led to a more comprehensive examination of patients. Newly detected co-morbidities and complications could be optimized pre-operatively to improve the clinical outcome. This is also evident considering the equal length of stay and reduced hours admitted to the ICU in the OGC cohort, despite the higher ASA scores and prolonged time to surgery. Nevertheless, other contributing factors might be the lack of ICU or operating theatre capacity.

Osteoporosis is among the most significant risk factors for fractures in older patients, particularly hip fractures, vertebral body fractures, and, increasingly, pelvic fractures [1]. Despite this knowledge, osteoporosis appears to remain underdiagnosed, and effective osteoporosis prophylaxis rarely is prescribed [42,43]. In our study, a significant improvement in osteoporosis therapy was achieved by establishing the GTC. Thus, 46.8% of the OGC cohort at discharge had vitamin D supplementation or specific osteoporosis therapy, compared to 13.1% of the UC cohort. As such, these results are comparable to previous studies, like that of Fisher et al., who recorded 43.7% receiving osteoporosis treatment at discharge after GTC establishment, but was significantly lower than the results of Cogan et al., with 54–60%, and Bücking et al., with 53–75% osteoporosis therapy at discharge [8,9,30]. This shows that, despite the progress already made, a further increase in osteoporosis prophylaxis and therapy should be addressed in our institution through more widespread awareness and teaching among physicians and staff members and more consistent implementation of the DGU’s existing SOP on the diagnostics and treatment of osteoporosis. 

Finally, the overall goal of ortho-geriatric treatment is to restore the patients´ physical function and autonomy [43]. Early postoperative mobilization contributes significantly by preserving muscle strength and reducing complications, like pneumonia or pressure ulcers [44]. By establishing the GTC, a significant increase in mobilization on the first postoperative day from 57.1% to 86.3% was achieved in our study. This exceeds the few published data on this topic, such as Bücking et al., reporting a mobilization of 58% on the first postoperative day in the context of ortho-geriatric co-management [30]. This may also contribute to the tendency towards a higher restoration of walking ability at discharge in our OGC cohort. While, in the UC cohort, only 52.4% were able to restore or improve their walking ability at discharge, 63.9% could do so in the OGC cohort. The positive effect of ortho-geriatric co-management on the patients’ mobility is well-known. In a four-month follow-up after surgical treatment of a femoral neck fracture in older people, Stenvall et al., for example, observed a recovery of walking ability in 62% with ortho-geriatric co-management compared to 49% in a traditional care model [13]. 

Our study has some limitations. First, as a retrospective cohort study, it depended on the electronic patient records and consistent documentation of comorbidities, complications, and test results. Due to the long observation period, changes in staff members, and medical definitions, documentation was partly inconclusive. Therefore, some information could not be obtained, or patients had to be excluded from the cohorts, limiting the validity of the study. A prospective controlled trial will be necessary to validate our findings. Second, there was no evaluation of long-term outcomes after discharge. Therefore, the results can only be considered as short-term results and interpreted as such. To evaluate the long-term outcomes of ortho-geriatric co-management, such as functional status, follow-up examinations will be required in the future. However, the one-year mortality of patients after hospitalization with fractures is not necessarily related to the acute trauma, and, therefore, has to be interpreted with caution. 

## 5. Conclusions

This retrospective cohort study shows that ortho-geriatric co-management is superior to usual care in terms of effectiveness and clinical outcome in patients with lumbar spine, pelvis, and acetabular fractures in a GTC DGU^®^. The establishment of a GTC DGU^®^ resulted in a higher number of detected comorbidities, such as urological complications, pneumonia, or delirium, as well as a reduced need for revision surgery, despite poorer baseline health conditions. Furthermore, the effectiveness of geriatric assessment and intervention was reflected in earlier mobilization, as well as higher rates of osteoporosis treatment at discharge. Moreover, this study provides a detailed overview of the important comorbidities of these patients through a comprehensive geriatric assessment and thus offers potential targets for demand-oriented patient care.

## Figures and Tables

**Table 1:** Demographic characteristics.

	UC (*n* = 137)	OGC (*n* = 224)	*p*-Value
Age in years, *n* (%)	81.5 (6.2)	82.2 (6.2)	0.566
Sex, *n* (%)			0.326
Male	35 (25.5)	68 (30,4)	
Female	102 (74.5)	156 (69.6)	
BMI, mean (SD)	25.9 (4.5)	24.9 (4.5)	0.089
ASA III/IV, *n* (%)	94 (68.8)	169 (75.4)	**0.002**
ACCI, mean (SD)	5.8 (1.5)	6.0 (1.4)	0.144
Fracture type, *n* (%)			**0.006**
Lumbar spine	55 (40.1)	86 (38.4)	0.764
Pelvic ring	51 (37.2)	98 (43.8)	0.230
Acetabulum	22 (16,1)	39 (17,4)	0.765
Others	9 (6.6)	1 (0.4)	**< 0.001**

ASA, American Society of Anesthesiologists. ACCI, age-adjusted Charlson Comorbidity Index. BMI, body mass index (kg/m^2^). OGC, ortho-geriatric co-management. UC, usual care. Data are expressed in absolute *n* and relative (%) frequencies or mean ± standard deviation (SD). Significant differences are indicated in bold type.

**Table 2:** Geriatric assessment and co-management.

Assessment		
Test	*n* (%)	Mean (SD)
ISAR	196 (87.5)	2.9 (1.7)
BI	146 (65.2)	39.2 (16.5)
mod. BI	79 (35.3)	71.3 (21.7)
MoCA	30 (13.4)	22.9 (4.4)
DemTect	39 (17.4)	6.9 (4.2)
GDS	95 (42.4)	3.6 (2.4)
MNA	109 (48.7)	21.2 (4.4)
DEMMI	72 (32.1)	23.4 (10.2)
**Co-Management**		
Treated by geriatrician, *n* (%)	122 (54.5)	
Hours to first contact, mean (SD)	95.9 (70.5)	

**Table 3:** Primary outcome measures.

Complication, *n* (%)	UC (*n* = 137)	OGC (*n* = 224)	*p*-Value
All	80 (58.4)	150 (67.0)	0.100
Urological	35 (25.5)	84 (37.5)	**0.021**
Electrolyte disorder	45 (32.8)	66 (29.5)	0.499
Anemia	36 (26.3)	47 (21.0)	0.246
Transfusion	29 (21.2)	40 (17.9)	0.438
Pulmonary	17 (12.4)	40 (17.9)	0.072
Delirium	9 (6.6)	27 (12.1)	0.091
Cardiac	12 (8.8)	18 (8.0)	0.405
Gastrointestinal	6 (4.4)	11 (4.9)	0.387
Surgical	3 (2.2)	12 (5.4)	0.143
Revision surgery	8 (5.8)	7 (3.1)	**0.012**
Neurologic	7 (5.1)	5 (2.2)	0.475
Dehydration	4 (2.9)	6 (2.7)	1.000
Acute kidney injury	1 (0.7)	6 (2.7)	0.260
In-hospital mortality	0	6 (2.7)	0.087
Sepsis/SIRS/Shock	1 (0.7)	5 (2.2)	0.415
Anesthesia	2 (1.5)	1 (0.4)	0.560
In-hospital fall	0	3 (1.3)	0.292

OGC, ortho-geriatric co-management. UC, usual care. Data are expressed in absolute *n* and relative (%) frequencies. Significant differences are indicated in bold type.

**Table 4:** Secondary outcome measures.

	UC (*n* = 137)	OGC (*n* = 224)	*p*-Value
Therapy regimen, *n* (%)			0.255
Surgery	83 (60.6)	122 (54.5)	
Conservative	54 (39.4)	102 (45.5)	
Length of stay in days, mean (SD)	10.4 (5.3)	10.4 (8.2)	0.111
Time to surgery in hours, mean (SD)	72.4 (54.5)	112.2 (75.8)	**< 0.001**
Hours at ICU, mean (SD)	14.3 (47.4)	11.2 (36.0)	0.660
Suspected dementia, *n* (%)	1 (0.7)	13 (5.8)	**0.022**
Pressure ulcer at discharge, *n* (%)	16 (11.7) (*n* = 137)	19 (9.4) (*n* = 203)	0.490
Osteoporosis therapy at discharge, *n* (%)			**< 0.001**
None	119 (86.9)	117 (53.2)	
Vitamin D	13 (9.5)	96 (43.6)	
Specific therapy	5 (3.6)	7 (3.2)	
Mobilization, first day, *n* (%)	48 (57.1) (*n* = 84)	101 (86.3) (*n* = 117)	**< 0.001**
Change in walking ability, *n* (%)	−0.9 (1.4) (*n* = 61)	−0.6 (1.1) (*n* = 122)	0.252
−4	4 (6.6)	3 (2.5)	
−3	4 (6.6)	8 (6.6)	
−2	13 (21.3)	11 (9.0)	
−1	8 (13.1)	22 (18.0)	
±0	26 (42.6)	76 (62.3)	
+1	6 (9.8)	2 (1.6)	
Reduced	29 (47.6)	44 (36.1)	
Restored/Improved	32 (52.4)	78 (63.9)	

OGC, ortho-geriatric co-management. UC, usual care. Significant differences are indicated in bold type.

## Data Availability

The data that support the findings of this study are available from the corresponding author, T.H., upon reasonable request.

## Supplementary Materials

The following are available online at https://www.mdpi.com/article/10.3390/medicina57080794/s1, Table S1: Detailed Geriatric Assessment.
