# Supplementary material for: Care of Geriatric Patients with Lumbar Spine, Pelvic, and Acetabular Fractures before and after Certification as a Geriatric Trauma Center DGU®: A Retrospective Cohort Study
Source: Medicina (Kaunas). 2021 Jul 31;57(8):794. doi: 10.3390/medicina57080794 (PMC8398181; doi:10.3390/medicina57080794)
Supplement: Supplementary file 1 [file medicina-57-00794-s001.zip › medicina-1298459-supplementary.pdf]

Title: Care of geriatric patients with lumbar spine, pelvic, and acetabular fractures before and after certification as a Geriatric Trauma Center DGU®: a retrospective cohort study.

Authors: Tobias Hafner, Alina Kollmeier, Markus Laubach, Matthias Knobe, Frank Hildebrand, Miguel Pishnamaz

Journal: Medicina

Supplementary Materials

Table S1: Detailed Geriatric Assessment

Test	n (%)
ISAR	196 (87.5)
ISAR <2	49 (25.0)
ISAR ≥ 2	147 (75.0)
BI	146 (65.2)
BI 0-15	12 (8.3)
BI 20-35	66 (45.2)
BI 40-55	45 (30.8)
BI 60-75	20 (13.6)
BI 80-95	3 (2.1)
mod. BI	79 (35.3)
mod. BI 70-90	52 (65.8)
mod. BI 20-65	27 (34.2)
mod. BI 0-15	0
MoCA	30 (13.4)
MoCA ≥ 26	11 (36,7)
MoCA < 26	19 (63,3)
DemTect	39 (17.4)
DemTect 13-18	4 (10,3)
DemTect 9-12	8 (20,5)
DemTect < 8	27 (69,2)
GDS	95 (42.4)
GDS 0-5	79 (83.2)
GDS 6-10	14 (14.7)
GDS 11-15	2 (2.1)
MNA	109 (48.7)
MNA 24-30	34 (31.2)
MNA 17-23.5	58 (53.2)
MNA <17	17 (15,6)
DEMMI	72 (32.1)

Data in absolute **n** and relative (%) frequencies. **ISAR** Identification of Seniors at Risk, **BI** Barthel-Index, **mod. BI** modified Barthel-Index, **MoCA** Montreal Cognitive Assessment, **GDS** Geriatric Depression Scale, **DEMMI** De Morton Mobility Index, **MNA** Mini Nutritional Assessment
